# Newton–Raphson preconditioner for Krylov type solvers on GPU devices

**DOI:** 10.1186/s40064-016-2346-7

**Published:** 2016-06-21

**Authors:** Noriyuki Kushida

**Affiliations:** Vienna, Austria

**Keywords:** Newton–Raphson method, Variable preconditioner, Krylov type solvers, Approximated Hessian matrix, Finite element method, GPGPU

## Abstract

A new Newton–Raphson method based preconditioner for Krylov type linear equation solvers for GPGPU is developed, and the performance is investigated. Conventional preconditioners improve the convergence of Krylov type solvers, and perform well on CPUs. However, they do not perform well on GPGPUs, because of the complexity of implementing powerful preconditioners. The developed preconditioner is based on the BFGS Hessian matrix approximation technique, which is well known as a robust and fast nonlinear equation solver. Because the Hessian matrix in the BFGS represents the coefficient matrix of a system of linear equations in some sense, the approximated Hessian matrix can be a preconditioner. On the other hand, BFGS is required to store dense matrices and to invert them, which should be avoided on modern computers and supercomputers. To overcome these disadvantages, we therefore introduce a limited memory BFGS, which requires less memory space and less computational effort than the BFGS. In addition, a limited memory BFGS can be implemented with BLAS libraries, which are well optimized for target architectures. There are advantages and disadvantages to the Hessian matrix approximation becoming better as the Krylov solver iteration continues. The preconditioning matrix varies through Krylov solver iterations, and only flexible Krylov solvers can work well with the developed preconditioner. The GCR method, which is a flexible Krylov solver, is employed because of the prevalence of GCR as a Krylov solver with a variable preconditioner. As a result of the performance investigation, the new preconditioner indicates the following benefits: (1) The new preconditioner is robust; i.e., it converges while conventional preconditioners (the diagonal scaling, and the SSOR preconditioners) fail. (2) In the best case scenarios, it is over 10 times faster than conventional preconditioners on a CPU. (3) Because it requries only simple operations, it performs well on a GPGPU. In addition, the research has confirmed that the new preconditioner improves the condition of matrices from a mathematical point of view by calculating the condition numbers of preconditioned matrices, as anticipated by the theoretical analysis.

## Background

Linear equation solvers require a great deal of computational time in many computer simulations, especially for large scale computing. Recently, Krylov type linear equation solvers have become quite common, because they require little memory space and exhibit fast convergence. In addition, Krylov type solvers can be implemented on highly parallel processing units like general purpose graphics processing units (GPGPU), which are expected to be one of the standards of the next generation high performance computing units. Krylov type solvers can be accelerated by using preconditioners. One of the most famous Krylov type solvers is the conjugate gradient method (CG). The CG method with preconditioner is sometimes called the preconditioned CG (PCG). Preconditioners transform the original linear system

1 $$\begin{aligned} \mathbf {Ax}=\mathbf {b} \end{aligned}$$

to

2 $$\begin{aligned} \mathbf {M}_1^{-1}\mathbf {AM}_2^{-1} \tilde{\mathbf {x}} = \tilde{\mathbf {b}}, \end{aligned}$$

where, $\mathbf {M}=\mathbf {M}_1 \mathbf {M}_2, \mathbf {M}_2 \mathbf {x}=\tilde{\mathbf {x}}, \mathbf {M}_1^{-1} \mathbf {b}= \tilde{\mathbf {b}}$, and $\mathbf {M}$ is a preconditioner, which represents $\mathbf {A}$ in some sense (Barrett et al. 1994; Meurant 2006). In the most extreme case, $\mathbf {M}$ is identical to $\mathbf {A}$, and therefore, the linear equation can be solved without any iterations. So far, no definitive preconditioner has been determined, thus developing preconditioner have been drawing attention from many researchers. Generally speaking, the basic strategies of constructing preconditioners are factorizing the original coefficient matrices to multiplied forms of diagonal matrices and triangular matrices, e.g. the incomplete lower upper factorization preconditioner (ILU), and approximating inverse of the coefficient matrices, e.g. sparse approximated inverse preconditioners (SPAI) (Benzi and Tûma 1999; Chow and Saad 1998). So far, several research groups have introduced preconditioned Krylov type solvers on GPGPU. However, only simple preconditioners or no preconditioner have been implemented (Cevahir et al. 2009; Georgescu and Okuda 2010), or powerful preconditioners do not perform well on GPGPU and CPU is used for preconditioning (Li and Saad 2013). At the same time, because there have been many reports about successful implementations on GPGPU, one can build a high performance linear equation solver with the help of programmers who have strong skills in code tuning using vast amount of time for implementation (Remmelg et al. 2016). However, most researchers cannot spare time for programming, and as such, an algorithm which can be used without difficulties may draw attention.

Preconditioners which are constructed through application of these principles must not change throughout Krylov solver iterations. Otherwise, the transformed systems will vary and thus either none of solver processes converge, or the obtained solutions are meaningless. On the other hand, several research groups have developed variable preconditioner for Krylov type solvers, which change the matrix elements of preconditioners in the Krylov solver iterations (Saad 1993; Aoto et al. 2010; van der Vorst and Vuik 1994; Chen et al. 2016). Variable preconditioners employ iterative solvers as preconditioners and continues iteration until the expected accuracy is obtained in preconditioning process. Practically, the transformation $\mathbf {M}_1^{-1} \mathbf {AM}_2^{-1}$ has never been performed, because this transformation results in a dense matrix although $\mathbf {A}$ is usually sparse. However, another linear equation $\mathbf {Mz}= \mathbf {r}$ is solved at each Krylov solver step, where $\mathbf {r}$ is the correction vector, and $\mathbf {z}$ is the correction vector in the transformed system. Therefore, we aimed to solve the new equation $\mathbf {Mz}= \mathbf {r}$ to perform preconditioning at each Krylov solver step, and to apply iterative solvers to the equation. One of the drawbacks of employing variable preconditioning is that only flexible variants of Krylov type solvers can be used with it. Nevertheless, fortunately, many flexible Krylov solvers have been developed e.g. generalized minimal residual method (GMRES) (Saad 1993), the generalized conjugate residual method (GCR) (Eisenstat et al. 1983), CG (Notay 2000), the quasi-minimal residual method (QMR) and the bi-conjugate gradient stabilized method (BiCGSTAB) (Chen et al. 2016).

On the other hand, one can show that Hessian matrices in the Newton–Raphson (NR) method can act as a preconditioner for the nonlinear conjugate gradient method (Kushida and Okuda 2004). In the literature, the researchers applied approximated Hessian matrices as preconditioners to the nonlinear conjugate gradient method to obtain the extremal value of the function

3 $$\begin{aligned} f_{nonlinear} (\mathbf {x})=\frac{ \left( \mathbf {x}^T \mathbf {A(x)x} \right) }{ \left( \mathbf {x}^T \mathbf {x} \right) } \end{aligned}$$

where $\mathbf {A}(\mathbf {x})$ is a matrix whose matrix elements vary according to the input vector $\mathbf {x}$. According to their article, the approximated Hessian preconditioner they developed was more suitable than conventional preconditioners like the diagonal scaling or the symmetric successive over relaxation method (SSOR). It should be stressed that they also pointed out that the CG method as a linear equation solver can be considered as a minimizer of the function

4 $$\begin{aligned} f_{linear} (\mathbf {x})=\frac{1}{2} \mathbf {x}^T \mathbf {Ax}- \mathbf {x}^T \mathbf {b} , \end{aligned}$$

where the vector $\mathbf {x}$ which gives the minimum value of the function corresponds to the solution of the linear system $\mathbf {Ax}=\mathbf {b}$ (Golub and Van Loan 1996). Therefore the Hessian matrix of the linear CG method corresponds to $\mathbf {A}$, we can expect approximated Hessian matrices will serve as good preconditioning matrices for the CG method and other Krylov type linear equation solvers. Obtaining either an approximated or full Hessian with reasonable computational cost remains a problem. However, fortunately, there has been a great deal of research with regard to construction of approximated Hessian matrices for quasi NR methods, e.g. the Broyden–Fletcher–Goldfarb–Shanno algorithm (BFGS), and its variants. Particularly, the limited memory BFGS (L-BFGS) algorithms indicate suitability as preconditioners because the algorithms use smaller amount of memory space and require less computational effort than full Hessian methods. In addition, the operations which are required in L-BFGS are quite simple i.e. vector dot product, scalar multiple of a vector, and vector addition. Those operations can be implemented with BLAS libraries, which are well optimized on many high performance computing units, and therefore, L-BFGS can be implemented without difficulties and expected to perform well even on a cutting-edge computer architecture like (Shobu supercomputer 2015). The BFGS method and its variants generate approximated Hessian matrices by modifying the matrices through NR iterations. Therefore, the flexible Krylov solvers only work well with BFGS approximated Hessians. Thus, in the current studies, the feasibility of BFGS approximated Hessian matrices as preconditioners with the GCR (a flexible Krylov solver) and the performance on a GPGPU device is examined as an example of highly parallel computing unit.

The developed algorithm is explained in the following “Methods” section. In the “Results and Discussion” section, the convergence behaviour of our new preconditioner as well as the performance on a GPGPU are investigated.

## Methods

In this section, we introduce our L-BFGS based preconditioner that works with GCR. First, we explain the BFGS and L-BFGS algorithms. Then, we introduce BFGS preconditioned GCR. Finally, L-BFGS preconditioned GCR is introduced.

### BFGS Hessian approximation

#### BFGS method

The BFGS method is prevalent as a quasi-Newton method (Kelley 1999). First we introduce the BFGS algorithms to show how the approximated Hessian is obtained. In Algorithm 1, $\mathbf {H}_k$ denotes the approximated Hessian matrix of a function $f(\mathbf {x}_k)$ at *k* th step, and $\nabla f(\mathbf {x}_k)$ denotes the gradient of $f(\mathbf {x}_k)$. $|| \mathbf {v}||$ denotes a vector norm of $\mathbf {v}$. The approximated Hessian matrix is updated on line 13. Approximations become better as the BFGS step continue.

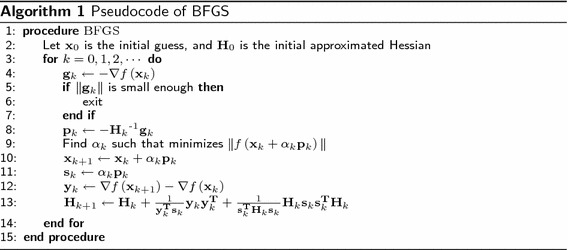



#### L-BFGS method

The BFGS method does not require exact Hessian matrices unlike the original Newton–Raphson method. It does, however, require huge amounts of memory. This is because Hessian matrix update operations rewrite all $\mathbf {H}$ elements. To reduce the amount of memory space required, a limited memory BFGS method (L-BFGS) was developed by Nocedal (1980). The algorithm was shown in Algorithm 2. In the L-BFGS algorithm, Hessian matrix updates are avoided, and the gradient vector is modified using vectors $\mathbf {y}$ and $\mathbf {s}$, which appear in the BFGS algorithm as well. Therefore, if the initial approximated Hessian matrix $\mathbf {H}_0$ is sparse and easily invertible, e.g. diagonal matrices, the L-BFGS method will not require large amounts of memory to store the Hessian matrix, in cases *m* is adequately small, where *m* denotes the number of previous iterations to be taken into consideration. The approximated Hessian matrix $\mathbf {H}_k$ is not updated in the algorithm but it need not be identical to the initial approximated Hessian matrix $\mathbf {H}_0$ at each time step.

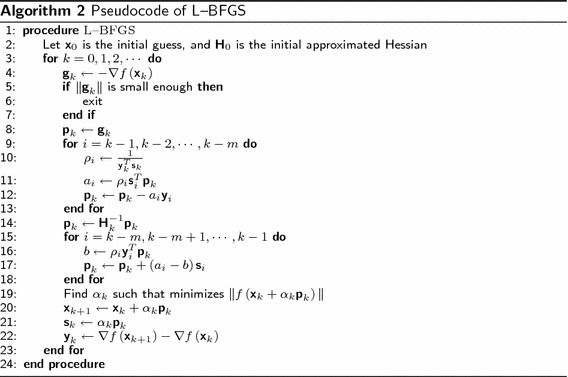



### Preconditioned GCR

#### GCR algorithm

The GCR method was developed by Eisenstat et al. (1983), and is a Krylov type linear equation solver. In their article, they developed the flexible version of the GCR, because the non-flexible version GCR requires $2 \times N \times N$ memory in addition to coefficient matrices. In these coefficient matrices *N* denotes the size of the coefficient matrix of the system being solved, to store Krylov sub-space basis. For this reason, practically, the full version GCR cannot be applied to large scale problems and the flexible version should instead be focused on. The algorithm of the flexible version GCR is indicated in Algorithm 3. In the algorithm, restart is applied every *n* step. Thus, only *n* basis vectors and *n* complimentary vectors ($\mathbf {q}$ and $\mathbf {p}$) need to be stored.

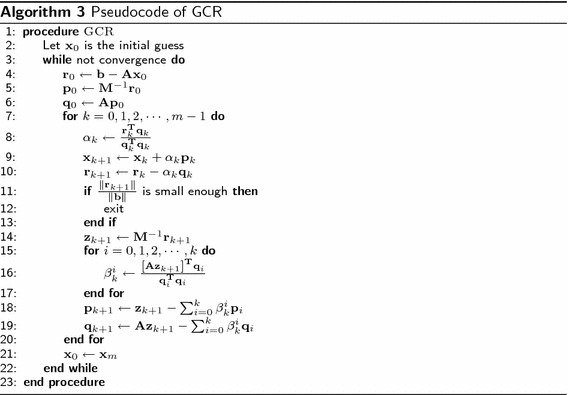



#### Conventional preconditioners

In this subsection, we introduce two conventional preconditioners, the diagonal scaling, and the SSOR. The preconditioner $\mathbf {M}$ of the diagonal scaling is defined as follows,

5 $$\begin{aligned} \mathbf {M}=diag(\mathbf {A}), \end{aligned}$$

where $diag(\mathbf {A})$ is the function which extracts the diagonal component from the matrix $\mathbf {A}$. The SSOR preconditioner is defined as follows,

6 $$\begin{aligned} \mathbf {M}(\omega )=\frac{1}{(2-\omega )} \left( \frac{1}{\omega } \mathbf {D}+ \mathbf {L} \right) \left( \frac{1}{\omega } \mathbf {D} \right) ^{-1} \left( \frac{1}{\omega } \mathbf {D}+\mathbf {U} \right) , \end{aligned}$$

where $\mathbf {D}$, $\mathbf {L}$, and $\mathbf {U}$ denote the diagonal, lower triangular, and upper triangular components of the coefficient matrix $\mathbf {A}$, respectively. In the current studies $\omega$ is set to 1.0.

### BFGS-preconditioned GCR

In this section, we will introduce the BFGS preconditioned GCR and the L-BFGS preconditioned GCR. As shown in Algorithm 1, the approximated Hessian matrix in the BFGS algorithm is updated with vectors $\mathbf {y}$ and $\mathbf {s}$, where $\mathbf {s}$ is the difference between the current iteration’s vector and the previous iteration, and $\mathbf {y}$ is the difference between the current iteration’s gradient vector and the previous iteration. Therefore, $\mathbf {s}$ and $\mathbf {y}$ at the *k* th step can be written as,

7 $$\mathbf {s}= \mathbf {x}_{k+1} - \mathbf {x}_k,$$

8 $$\mathbf {y}= \nabla f_{k+1} - \nabla f_k.$$

Now, we consider the function,

9 $$\begin{aligned} f (\mathbf {x})=\frac{1}{2} \mathbf {x}^T \mathbf {Ax}- \mathbf {x}^T \mathbf {b}. \end{aligned}$$

As previously described, the Hessian of the function is $\mathbf {A}$, and the gradient is

10 $$\begin{aligned} \nabla f(\mathbf {x})=\mathbf {Ax}-\mathbf {b}=- \mathbf {r}. \end{aligned}$$

Here it can be considered that if we perform a BFGS update with above vectors $\mathbf {s}$, $\mathbf {y}$ and $\mathbf {r}$, the obtained approximated Hessian matrix must be an approximation of $\mathbf {A}$ This is because we implicitly minimize the function when the gradient is defined as above. In addition, using the above relationships, vectors $\mathbf {s}$ and $\mathbf {y}$ can be obtained within the GCR algorithm, and therefore, the approximation of $\mathbf {A}$, the Hessian matrix of the function, can be obtained using the BFGS update within the GCR algorithm, and it can be used as a preconditioner. The GCR algorithm with the BFGS and the L-BFGS preconditioniners are described in the following subsections.

#### BFGS–GCR

First, we introduce the GCR with the full size Hessian BFGS. The algorithm is shown in Algorithm 4. In contrast to the conventional GCR algorithm, lines 5 to 9 are added, and the Hessian matrix is updated. In order to calculate vectors $\mathbf {s}$ and $\mathbf {y}$, the solution vector and the gradient vector just after restart are stored (line 12 and 13). Lines 14 to 27, are the same as the original GCR. We can liken them to finding $\alpha _k$ which appears at line 9 in the BFGS algorithm. This algorithm is useless from a practical viewpoint, in terms of both memory space and computational costs. This is because a dense $N \times N$ matrix must be managed.

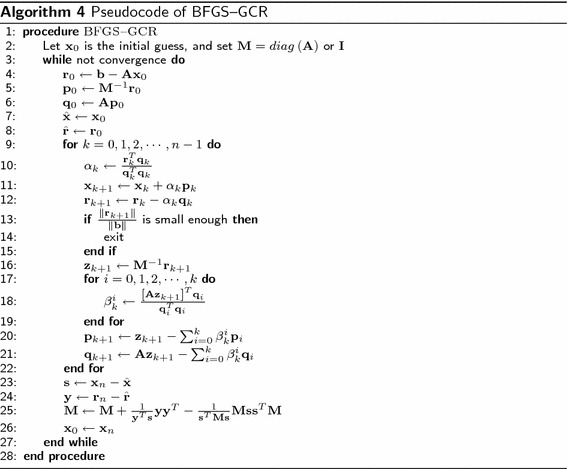



#### L-BFGS–GCR

Once the BFGS preconditioned GCR algorithm is obtained, obtaining the L-BFGS preconditioned GCR algorithm is quite straightforward. Algorithm 5 indicates the L-BFGS preconditioned GCR algorithm. In the algorithm {} denotes a set of vectors. The algorithm is similar to the BFGS–GCR algorithm. However, there are two main differences: (1) there is no Hessian update, and (2) the preconditioner system solution cannot be written down explicitly. Instead it is written as a procedure (lines 13, and 22. The procedures are indicated in Algorithm 6). In addition, vectors used for the L-BFGS procedure are stored (lines 8 and 9).

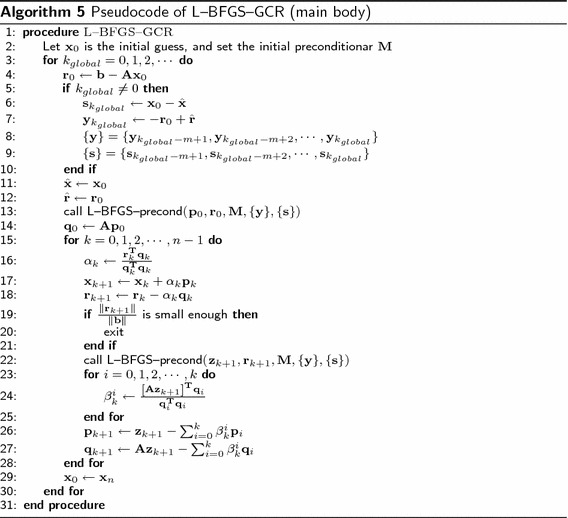

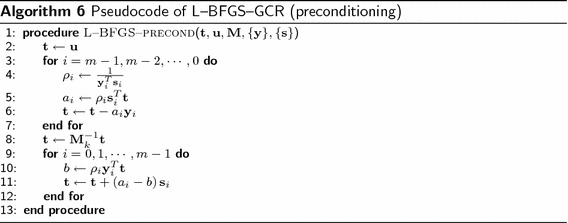





## Results and discussion

In order to check the feasibility of the BFGS preconditioners, we investigate the convergence rate of the GCR with conventional preconditioners and the BFGS preconditoners. In the current studies, the diagonal scaling and the SSOR are employed as conventional preconditioners, and the BFGS and the L-BFGS are newly developed preconditioners. In the cases of BFGS and L-BFGS, the choice of initial Hessian matrices remains an issue. In the current studies, the diagonal scaling preconditioner is employed as an initial Hessian matrix for the BFGS and the L-BFGS, and the SSOR preconditioner is employed for the L-BFGS as well. In addition, in the case of the L-BFGS, we must determine *m*, which is the number of previous iterations to be taken into consideration. Unfortunately, with our best knowledge, there is no obvious rule on the choice of *m* even for the L-BFGS as a nonlinear equation solver. Therefore, we investigate the convergence rate with various *m* i.e. $m = 3,5,7,$ and 10. The algorithms of each preconditioner are given in the “Methods” section in this article.

### Matrix market matrices

In this section, we employ matrices which can be obtained on the matrix market website, which collects a huge variety of sample matrices (Boisvert et al. 1997, http://math.nist.gov/MatrixMarket/). The properties of employed matrices are listed in Table 1.

**Table 1:** Properties of matrices used in the current studies

	Type	N	NNZ
GR_30_30	SPD: Finite-difference Laplacians	900	7742
BCSSTK14	SPD: Structural Engineering Matrices	1806	63,454
BCSSTK15	SPD: Structural Engineering Matrices	3948	117,816
RDB450	Unsymmetric: Reaction–diffusion Brusselator Model	450	2580

N denotes the dimension of a matrix, and NNZ denotes the number of nonzero components. SPD stands for symmetric positive definite

In the table, SPD is symmetric positive definite, N shows the dimension of each matrix, and NNZ shows the number of nonzero components. GR_30_30, BCSSTK14, and BCSSTK15 are all SPD. RDB450 is unsymmetric but we performed the test with it in order to check the feasibility of the BFGS preconditioners on a non-SPD matrix. The numbers of iterations to convergence, the time to convergence, and the solution error of each preconditioner are listed (Tables 2, 3, 4).The convergence criteria is that the relative residual norm $\Vert \mathbf {r} \Vert _2/\Vert \mathbf {b} \Vert _2 < 10^{-8}$, where $\Vert \Vert _2$ denotes 2-norm of a vector. In the tables, the number of Krylov sub-space for the GCR is fixed $(n=10)$, and NA shows that the GCR with a preconditioner does not converge within 150,000 iterations. In addition, DIAG means the diagonal scaling preconditioner, SSOR means the SSOR preconditioner, BFGS–DIAG means the L-BFGS preconditioner with DIAG as the initial Hessian, BFGS–SSOR means the L-BFGS preconditioner with SSOR as the initial Hessian, and BFGS means the BFGS preconditioner. The time to convergence for the BFGS preconditioner is not recorded, because the BFGS preconditioner requires lower–upper (LU) factorization and practically meaningless. The computer environment is as follows: OS: Scientific Linux6, CPU: AMD E-350, RAM:4Gbyte, Compiler: Intel C compiler 13.1.1, Lapack & BLAS: Intel MKL 11.0.3.

**Table 2:** Numbers of GCR iterations to convergence

	DIAG	SSOR	BFGS–DIAG				BFGS–SSOR				BFGS
			m = 3	m = 5	m = 7	m = 10	m = 3	m = 5	m = 7	m = 10	
GR_30_30	224	55	155	120	157	169	47	50	50	50	65
BCSSTK14	2422	NA	1554	1447	1415	1668	439	535	526	584	584
BCSSTK15	NA	NA	5448	7161	5856	NA	1599	1474	2396	NA	788
RDB450	NA	NA	113,940	111,350	86,928	NA	15,066	19,880	19,960	26,308	NA

NA denotes the preconditioned GCR doesn’t converge within 150,000 iterations, and m denotes the number of previous iterations to be taken into consideration

**Table 3:** Error in solutions

	DIAG	SSOR	BFGS–DIAG				BFGS–SSOR				BFGS
			m = 3	m = 5	m = 7	m = 10	m = 3	m = 5	m = 7	m = 10	
GR_30_30	1.14E−08	9.28E−09	5.06E−09	4.13E−09	4.52E−09	5.22E−09	3.46E−09	2.12E−09	2.12E−09	2.12E−09	1.60E−04
BCSSTK14	1.44E−12	NA	1.01E−10	6.29E−12	1.42E−12	1.85E−12	5.59E−12	1.19E−11	1.37E−12	1.83E−12	5.90E−09
BCSSTK15	NA	NA	6.69E−11	1.10E−10	1.03E−10	NA	1.05E−11	9.53E−12	1.22E−11	NA	1.51E−08
RDB450	NA	NA	1.34E−08	1.44E−08	1.22E−08	NA	9.93E−09	1.12E−08	1.52E−08	1.27E−08	NA

In the table, e.g. 1.14E−08 means that the error defined as $\Vert \mathbf {x}_{true}-\mathbf {x}_{GCR} \Vert _2$ is $1.14 \times 10^{-8}$, where $\mathbf {x}_{true}$ is the solution by the LU factorization, and $\mathbf {x}_{GCR}$ is the solution by the preconditioned GCR. NA denotes the preconditioned GCR doesn’t converge within 150,000 iterations, and m denotes the number of previous iterations to be taken into consideration

**Table 4:** Time to convergence

	DIAG	SSOR	BFGS–DIAG				BFGS–SSOR			
			m = 3	m = 5	m = 7	m = 10	m = 3	m = 5	m = 7	m = 10
GR_30_30	5.14E−02	1.50E−02	4.07E−02	3.43E−02	4.86E−02	5.73E−02	1.40E−02	1.56E−02	1.56E−02	1.56E−02
BCSSTK14	2.95E+00	NA	2.02E+00	1.97E+00	2.01E+00	2.54E+00	7.55E−01	9.60E−01	9.83E−01	1.15E+00
BCSSTK15	NA	NA	1.44E+01	2.00E+01	1.73E+01	NA	5.46E+00	5.29E+00	8.97E+00	NA
RDB450	NA	NA	1.33E+01	1.46E+01	1.27E+01	NA	2.06E+00	3.01E+00	3.30E+00	4.94E+00

In the table, e.g. 5.14E−02 means $5.14 \times 10^{-2}$ s. NA denotes the preconditioned GCR doesn’t converge within 150,000 iterations, and m denotes the number of previous iterations to be taken into consideration

In the current studies, BFGS–SSOR shows the best convergence rate in all cases except BCSSTK15. BFGS shows the similar performance as BFGS–SSOR, but it does not converge in RDB450 case. BFGS–DIAG is worse than BFGS–SSOR and BFGS. However, with the best selection of *m*, it converges when DIAG and SSOR do not converge i.e. in the cases of BCSSTK15 and RDB450 when $m=10$ BFGS–DIAG does not converge, but when $m=3,5$, and 7 it converges in all cases, even when DIAG and SSOR do not converge (BCSSTK15 and RDB450). Therefore, it can be said that BFGS preconditioners are more robust than conventional preconditioners.

In terms of time to convergence, BFGS–SSOR shows the best performance as well, and the more problems are difficult to solve, the better performance BFGS–SSOR shows.

The solution error we used is defined as $\Vert \mathbf {x}_{true}-\mathbf {x}_{GCR} \Vert _2$, where $\mathbf {x}_{true}$ is the solution obtained using Lapack LU factorization routine, and $\mathbf {x}_{GCR}$ is the solution obtained using GCR with preconditioners. Except BFGS with GR_30_30, the errors are smaller than $O(10^{-8})$, therefore, it can be said that the BFGS and the L-BFGS preconditioners do not cause problems on solution.

**Fig. 1:**
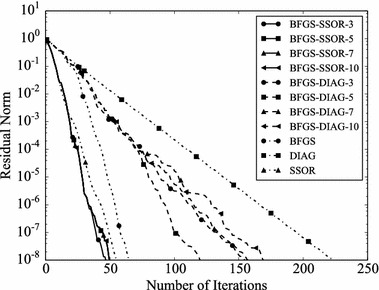
Convergence histories of each preconditioner on GR_30_30. The *vertical axis* shows the residual norm $\Vert \mathbf {r} \Vert _2 / \Vert \mathbf {b} \Vert _2$ , and the *horizontal axis* shows the number of iterations

**Fig. 2:**
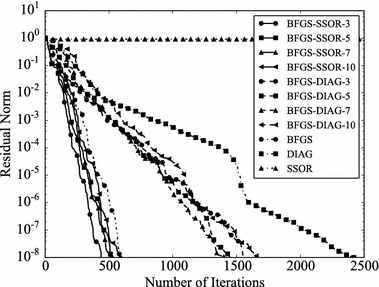
Convergence histories of each preconditioner on BCSSTK14. The *vertical axis* shows the residual norm $\Vert \mathbf {r} \Vert _2 / \Vert \mathbf {b} \Vert _2$, and the *horizontal axis* shows the number of iterations. The plot is truncated at the 2500th iteration

**Fig. 3:**
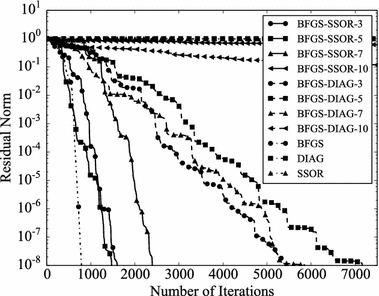
Convergence histories of each preconditioner on BCSSTK15. The *vertical axis* shows the residual norm $\Vert \mathbf {r} \Vert _2 / \Vert \mathbf {b} \Vert _2$, and the *horizontal axis* shows the number of iterations. The plot is truncated at the 7500th iteration

**Fig. 4:**
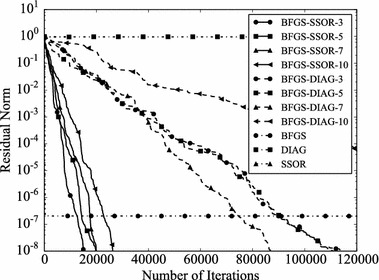
Convergence histories of each preconditioner on RDB450. The *vertical axis* shows the residual norm $\Vert \mathbf {r} \Vert _2 / \Vert \mathbf {b} \Vert _2$, and the *horizontal axis* shows the number of iterations. The plot is truncated at the 120,000th iteration

Convergence histories of preconditioners on each problem are plotted (Figs. 1, 2, 3, 4). In the figures, the relative residual norm $\Vert \mathbf {r} \Vert _2 / \Vert \mathbf {b} \Vert _2$ at each iteration is plotted. Generally, the bigger the number of iterations becomes, the better convergence rate is obtained. This tendency can be clearly seen in Fig. 1. In the figure, DIAG and BFGS–DIAG show the similar convergence rate at the beginning (up to 20th iteration). However, around 40th iteration, BFGS–DIAG shows better convergence rate than DIAG. The same behaviour can be seen on BFGS, whose initial Hessian matrix corresponds to DIAG. The reason of the acceleration is that the approximation of the Hessian matrix becomes better when the GCR iteration continues. The curves of BFGS–SSOR are steeper than those of BFGS–DIAG. These results give the following insight: the convergence rates of BFGS like methods depend on the degrees of approximation of initial Hessian matrices. In contrast, there is the case which the BFGS preconditioner does not converge. This result can be seen in the RDB450 case. In that case, BFGS cannot improve the solution after the residual norm reaches around $2 \times 10^{-7}$. It is considered that the behavior is caused by the poor approximation accumulates errors when the Hessian matrix is updated. On the other hand, thanks to the effect of using only the latest results, BFGS–DIAG and BFGS–SSOR converge, when BFGS does not converge. In other words, the L-BFGS preconditioner is more robust than the BFGS preconditioner.

### Large and Ill-posed problems

**Fig. 5:**
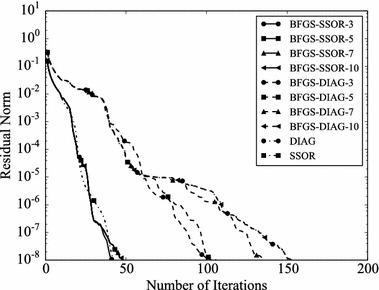
Convergence histories of each preconditioner on the $25^{3}$ problem. The *vertical axis* shows the residual norm $\Vert \mathbf {r} \Vert _2 / \Vert \mathbf {b} \Vert _2$, and the *horizontal axis* shows the number of iterations

**Fig. 6:**
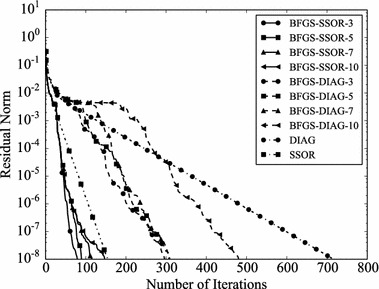
Convergence histories of each preconditioner on the $50^{3}$ problem. The *vertical axis* shows the residual norm $\Vert \mathbf {r} \Vert _2 / \Vert \mathbf {b} \Vert _2$, and the *horizontal axis* shows the number of iterations

**Fig. 7:**
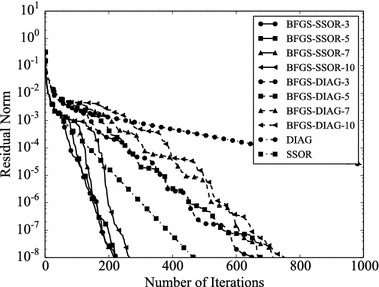
Convergence histories of each preconditioner on the $100^{3}$ problem. The *vertical axis* shows the residual norm $\Vert \mathbf {r} \Vert _2 / \Vert \mathbf {b} \Vert _2$, and the *horizontal axis* shows the number of iterations. The plot is truncated at the 1000th iteration

In this section, we perform a performance investigation with matrices generated with finite element method (FEM) for solving Poisson’s equation. This is because matrices in the matrix market are small for nowadays computers. The physical analysis domain is a cubic whose edge length is 1.0. Dirichlet boundary conditions are given on the both faces perpendicular to *z*-axis (0 and 100 degrees celsius respectively). The analysis domain is descritized with the first order 8 noded elements. In this section, the investigation is not carried out with BFGS-GCR, because of the shortage of memory space. First, we consider matrices descritized with cubic elements. In the present studes, we prepared $25^3$ degree of freedom (DOF), $50^3$ DOF, and $100^3$ DOF problems. The number of iterations to convergence, and time to convergence are listed in Tables 5 and 6. The effect of the L-BFGS preconditioner becomes better when DOF becomes larger. The convergence histories of each preconditioner on $25^3$, $50^3$, and $100^3$ DOF problems are shown in Figs. 5, 6, and 7. Generally, the BFGS preconditioners have steeper curves than the conventional preconditioners, and therefore, converge faster.

**Table 5:** Numbers of GCR iterations to convergence on large problems

	DIAG	SSOR	BFGS–DIAG				BFGS–SSOR			
			m = 3	m = 5	m = 7	m = 10	m = 3	m = 5	m = 7	m = 10
$25^3$	153	43	104	106	136	153	43	48	48	48
$50^3$	716	155	309	296	308	480	79	90	116	148
$100^3$	2557	467	653	680	716	754	224	220	216	265

In the table, m denotes the number of previous iterations to be taken into consideration

**Table 6:** Time to convergence on large problems

	DIAG	SSOR	BFGS–DIAG				BFGS–SSOR			
			m = 3	m = 5	m = 7	m = 10	m = 3	m = 5	m = 7	m = 10
$25^3$	9.45E−01	2.96E−01	5.06E−01	5.56E−01	7.65E−01	9.37E−01	3.14E−01	3.54E−01	3.57E−01	3.54E−01
$50^3$	2.93E+01	9.41E+00	1.46E+01	1.57E+01	1.79E+01	3.16E+01	5.19E+00	6.28E+00	8.53E+00	1.17E+01
$100^3$	9.68E+02	2.57E+02	2.84E+02	3.31E+02	3.78E+02	4.55E+02	1.35E+02	1.42E+02	1.48E+02	1.97E+02

In the table, e.g. 5.14E−02 means $5.14 \times 10^{-2}$ s, and m denotes the number of previous iterations to be taken into consideration

**Fig. 8:**
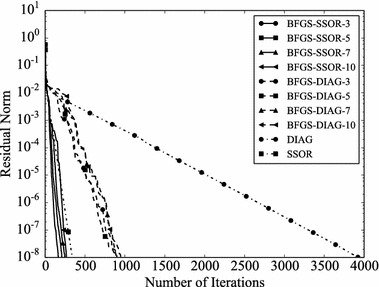
Convergence histories of each preconditioner on the $40 \times 40 \times 125$ problem. The *vertical axis* shows the residual norm $\Vert \mathbf {r} \Vert _2 / \Vert \mathbf {b} \Vert _2$, and the *horizontal axis* shows the number of iterations

**Fig. 9:**
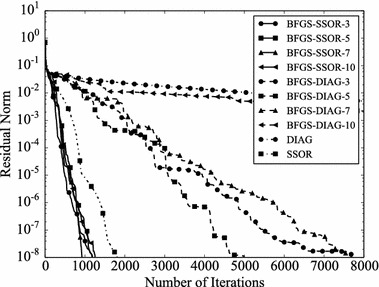
Convergence histories of each preconditioner on the $20 \times 20 \times 500$ problem. The *vertical axis* shows the residual norm $\Vert \mathbf {r} \Vert _2 / \Vert \mathbf {b} \Vert _2$, and the *horizontal axis* shows the number of iterations. The plot is truncated at the 8000th iteration

**Fig. 10:**
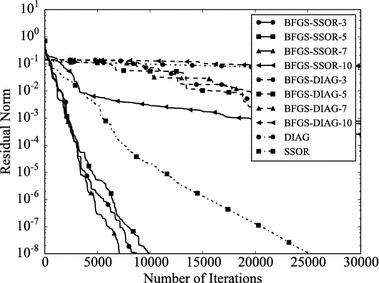
Convergence histories of each preconditioner on the $10 \times 10 \times 2000$ problem. The *vertical axis* shows the residual norm $\Vert \mathbf {r} \Vert _2 / \Vert \mathbf {b} \Vert _2$, and the *horizontal axis* shows the number of iterations. The plot is truncated at the 30,000th iteration

Next, we consider ill-posed problems. In FEM, the high aspect ratio elements generates ill-posed problems (Kamenski and Huang 2013). In the present studies, we prepare three problems by changing the number of elements along *z*-axis i.e. DOFs along *x*, *y*, and *z* axes are $40 \times 40 \times 125$, $20 \times 20 \times 500$, and $10 \times 10 \times 2000$. Obviously the total DOFs of each problem are identical ($2 \times 10^{5}$). The number of iterations to convergence, and times to convergence are listed in Tables 7 and 8. In the tables, computations are stopped if the residual norms do not meet the convergence criteria ($10^{-8}$) within 300,000 GCR iterations. The effect of the L-BFGS preconditioner becomes better when the problems become more ill-posed. The convergence histories of each preconditioner on $40 \times 40 \times 125$, $20 \times 20 \times 500$, and $10 \times 10 \times 2000$ DOF problems are shown in Figs. 8, 9, and 10. As observed in the previous section, the BFGS preconditioners have steeper curves than the conventional preconditioners, and therefore, converge faster. In addition, L-BFGS–SSOR constantly reduces residual norms, while the other preconditioners decrease their convergence speeds.

**Table 7:** Numbers of GCR iterations to convergence on ill-posed problems

	DIAG	SSOR	BFGS–DIAG				BFGS–SSOR			
			m = 3	m = 5	m = 7	m = 10	m = 3	m = 5	m = 7	m = 10
$40 \times 40 \times 125$	3930	341	878	900	964	891	165	210	248	267
$20 \times 20 \times 500$	NA	1817	7709	4994	7690	NA	1188	1187	926	1256
$10 \times 10 \times 2000$	NA	25,209	NA	NA	NA	NA	8736	9954	7088	NA

In the table, NA denotes the preconditioned GCR doesn’t converge within 300,000 iterations, and m denotes the number of previous iterations to be taken into consideration

**Table 8:** Time to convergence on ill-posed problems

	DIAG	SSOR	BFGS–DIAG				BFGS–SSOR			
			m = 3	m = 5	m = 7	m = 10	m = 3	m = 5	m = 7	m = 10
$40 \times 40 \times 125$	2.91E+02	3.62E+01	7.43E+01	8.57E+01	1.02E+02	1.08E+02	1.87E+01	2.58E+01	3.28E+01	3.81E+01
$20 \times 20 \times 500$	NA	1.87E+02	6.25E+02	4.58E+02	7.87E+02	NA	1.34E+02	1.46E+02	1.24E+02	1.84E+02
$10 \times 10 \times 2000$	NA	2.43E+03	NA	NA	NA	NA	9.32E+02	1.18E+03	9.09E+02	NA

In the table, e.g. 5.14E−02 means $5.14 \times 10^{-2}$ s, NA denotes the preconditioned GCR doesn’t converge within 300,000 iterations, and m denotes the number of previous iterations to be taken into consideration

### Performance on GPGPU

So far, we have confirmed that our new preconditioner improves the convergence rate of GCR. In this section, we implement it on GPGPU and investigate the performance. The matrices are the same as the previous section, but the problems are $100^3$ DOF and $200^3$ DOF. The computational environment is as follows: CPU:AMD A8-3850, RAM:32GB, GPU:Radeon R390 with 8GB RAM, OS: Ubuntu 14.04, Compiler: GCC-4.8.4, OpenCL compiler: AMDAPPSDK-3.0.0 beta. In the present study, DIAG and BFGS–DIAG are implemented on GPGPU, because SSOR cannot be implemented (Kushida and Okuda 2008). The matrices are stored with the ELL format on GPGPU, while they are stored with the CRS format on CPU (Barrett et al. 1994). Most operations are implemented with clBLAS, which provides users with BLAS functions on GPGPU and can be expected perform well (clBLAS 2015).

The time to convergence, the numbers of iterations to convergence on GPGPU with $n=5$, $m=3$ are shown in Tables 9 and 10 respectively. In the tables, the results of SSOR preconditioned GCR is also shown. In all cases, BFGS–DIAG shows the best perfornce in terms of both computational time and convergence rate. BFGS–DIAG is over four times faster than SSOR in the $200^3$ case (BFGS–DIAG:$9.50 \times 10^2$ s, SSOR:$3.88 \times 10^3$ s). On the other hand, DIAG is two times slower than SSOR, even it is on GPU (DIAG:$8.23 \times 10^3$ s, SSOR:$3.88 \times 10^3$ s).

**Table 9:** Time to convergence of GCR for each preconditioner on GPGPU and CPU

	DIAG (GPU)	BFGS–DIAG (GPU)	SSOR (CPU)
$100^3$	2.89E+02	5.27E+01	1.46E+02
$200^3$	8.23E+03	9.50E+02	3.88E+03

In the table, e.g. 5.14E−02 means $5.14 \times 10^{-2}$ s

**Table 10:** Numbers of GCR iterations for each preconditioner on GPGPU and CPU

	DIAG (GPU)	BFGS–DIAG (GPU)	SSOR (CPU)
$100^3$	4267	579	579
$200^3$	14,935	1293	1802

The average times per iteration are shown in Table 11. The GPU implementations show almost three times better performance than CPU. Our implementation relies on clBLAS, and further improvement can be expected with the improvement of clBLAS. On the other hand, by comparing DIAG and BFGS–DIAG, it can be calculated that the computational effort for L-BFGS corresponds to 40 % of the entire GCR algorithm. In the literature (Li and Saad 2013), powerful preconditioners do not always perfom well and they were operated on CPU instead of GPU. Therefore, it can be said that our new preconditioner is as powerful as conventional preconditioners, and perform better than conventional ones on GPU.

**Table 11:** Average computational time per iteration for each preconditioner on GPGPU and CPU

	DIAG (GPU)	BFGS–DIAG (GPU)	SSOR (CPU)
$100^3$	6.77E−02	9.10E−02	2.52E−01
$200^3$	5.51E−01	7.35E−01	2.15E+00

In the table, e.g. 5.14E−02 means $5.14 \times 10^{-2}$ s

### Discussion

#### Condition number

The convergence rate of Krylov type solvers depends on the conditions of coefficient matrices. The condition number is often used to indicate the conditioning of matrices. In other words, if a preconditioner is effective, the condition number of the preconditioned coefficient matrix should be better than the original one (Kushida 2015). The condition number is defined as,

11 $$\begin{aligned} \epsilon = \Vert \mathbf {A} \Vert \Vert \mathbf {A}^{-1} \Vert , \end{aligned}$$

where $\Vert \mathbf {A} \Vert$ denotes a norm of a matrix $\mathbf {A}$. In the case the norm is defined by 2-norm and if $\mathbf {A}$ is SPD, the condition number is given by,

12 $$\begin{aligned} \epsilon =\frac{(maximum \ eigenvalue \; of \; \mathbf {A})}{(minimum \; eigenvalue \; of \; \mathbf {A})}. \end{aligned}$$

**Fig. 11:**
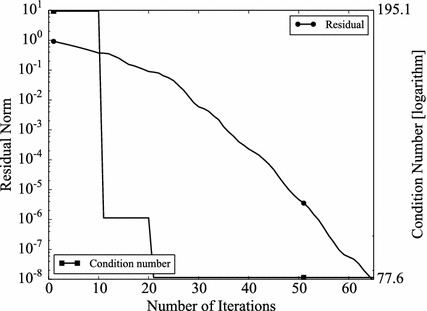
Histories of condition number and residual norm of BFGS–GCR on GR_30_30. The *left vertical axis* shows the residual norm $\Vert \mathbf {r} \Vert _2 / \Vert \mathbf {b} \Vert _2$, the *right vertical axis* shows the condition number in logarithmic scale and the *horizontal axis* shows the number of iterations

**Fig. 12:**
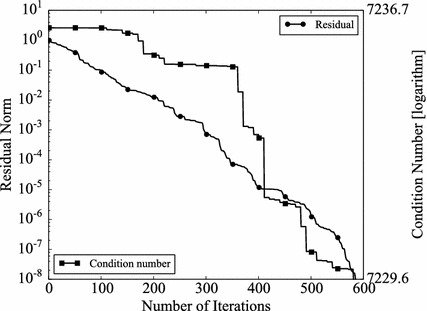
Histories of condition number and residual norm of BFGS–GCR on BCSSTK14. The *left vertical axis* shows the residual norm $\Vert \mathbf {r} \Vert _2 / \Vert \mathbf {b} \Vert _2$, the *right vertical axis* shows the condition number in logarithmic scale and the *horizontal axis* shows the number of iterations

**Fig. 13:**
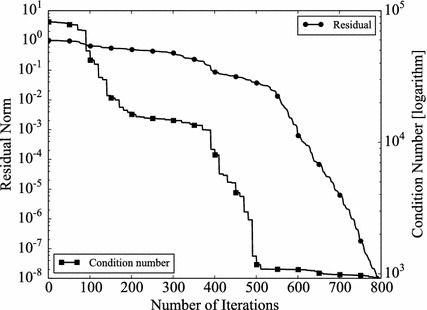
Histories of condition number and residual norm of BFGS–GCR on BCSSTK15. The *left vertical axis* shows the residual norm $\Vert \mathbf {r} \Vert _2 / \Vert \mathbf {b} \Vert _2$, the *right vertical axis* shows the condition number in logarithmic scale and the *horizontal axis* shows the number of iterations

Obviously, $\epsilon \ge 1$, and the smaller $\epsilon$, the better matrices are conditioned. If $\mathbf {A}$ is SPD, $\mathbf {A}$ can be decomposed by Cholesky decomposition i.e.

13 $$\begin{aligned} \mathbf {A}=\mathbf {LL}^T, \end{aligned}$$

where $\mathbf {L}$ is a lower triangular matrix. The BFGS update always gives SPD matrices, when the initial Hessian matrices are SPD. Therefore, in the GR_30_30, BCSSTK14, and BCSSTK15 case, the BFGS preconditioner always can be decomposed. Thus, the preconditioned coefficient matrix $\tilde{\mathbf {A}}$ can be obtained as,

14 $$\begin{aligned} \tilde{\mathbf {A}} =\mathbf {P}^{-1} \mathbf { AP}^{-T}, \end{aligned}$$

where, $\mathbf {P}$ is the Cholesky decomposed matrix $\mathbf {PP}^T = \mathbf {M}$, and $\mathbf {M}$ is the Hessian matrix, which appears in BFGS–GCR. Since the preconditioner in L-BFGS–GCR is not explicitly calculated and thus obtaining preconditioned coefficient matrices is difficult, we focus on BFGS–GCR in the rest of this section. However, L-BFGS shares the same mathematical background with BFGS. Therefore, the discussion with regard to BFGS can be applied to L-BFGS. The condition numbers of preconditioned matrices for GR_30_30, BCSSTK14, BCSSTK15 are shown with that of the original matrices (Table 12). Since the preconditioner varies through the BFGS–GCR iterations, the numbers at the first and the last step are shown. As expected, BFGS preconditioner becomes more effective when the BFGS–GCR iteration proceeds (GR_30_30: Condition number changes from $1.95 \times 10^2$ to$7.81 \times 10^1$, BCSSTK14: from $7.24 \times 10^3$ to $7.23 \times 10 ^ 3$, BCSSTK15: from $8.21 \times 10^4$ to $9.23 \times 10^2$). The condition number histories are plotted in Figs. 11, 12 and 13. In the figures, the residual norms are plotted as well. In all cases, the more BFGS–GCR iteration continues, the better condition numbers become. There is no obvious relationship between convergence rate and condition number in the GR_30_30, and BCSSTK14 cases. This is because, the condition numbers are improved but the differences are small. On the other hand, in the BCSSTK15 case, the acceleration in convergence rate is observed shortly after the condition number is improved (from 400th to 500th step, condition number is improved by around 10 times, and convergence rate is improved from 500th step). Consequently, it can be said that the BFGS preconditioner, and therefore the L-BFGS preconditioners as well, improve the condition of coefficient matrices as conventional preconditioners do.

**Table 12:** Condition numbers of original and BFGS preconditioned matrices

	Condition number		
	First	Last	original
GR_30_30	$1.95 \times 10^2$	$7.81 \times 10^1$	$1.95 \times 10^2$
BCSSTK14	$7.24 \times 10^3$	$7.23 \times 10^3$	$1.19 \times 10^{10}$
BCSSTK15	$8.21 \times 10^4$	$9.23 \times 10^2$	$6.54 \times 10^9$

Since BFGS preconditioner varies through GCR iterations, the condition numbers at the first and the last steps are shown

#### Performance estimation

As explained in the “Methods” section, the L-BFGS preconditioning can be achieved with two vector operations (Algorithm 6): one is the vector dot product, and the other is the addition of two vectors. Those operations can be implemented with BLAS functions: DDOT and DAXPY, respectively. Highly optimized BLAS libraries are usually available on high performance computers. AMD’s clBLAS is one of such BLAS libraries for AMD’s GPGPU devices, and we employed it to build the working program in the present article. The performance of the above BLAS functions depend on the memory bandwidth of the GPGPU device, because the number of arithmetic operations per double precision word fetch is one for both operations, while the theoretical performance of our device is 13 arithmetic operations per double precision word fetch. Therefore, it is safe to assume that the performances of those BLAS functions are proportional to the bandwidths of devices as the first estimation. On the other hand, Table 11 shows that the ratio of computational times of the GCR with two preconditioners is 1.33 : 1.0 in both $100^3$ and $200^3$ problems, and the difference originates in additional operations for our developed preconditioner. Now we assume that the change in the GPGPU memory bandwidth only affects the performance of our L-BFGS preconditioner, in other words, if the bandwidth becomes half, the ratio of computational times becomes 1.66 : 1.0. This is the severest assumption for the preconditioner, because the performance of other parts of the GCR must be deteriorated with lower bandwidth. With this assumption, we can estimate the lowest bandwidth with which our L-BFGS preconditioner converges faster than the diagonal scaling, and we obtain the following equation:

15 $$\begin{aligned} \left( T_{DIAG1iter} + T_{L-BFGSprecond} \times BWR \right) \times N_{ITERlbfgs}= T_{DIAG converge}, \end{aligned}$$

where, $T_{DIAG1iter}$ is the computational time per iteration of the GCR with the diagonal scaling, $T_{L\text {-}BFGSprecond}$ is computational time per iteration of the L-BFGS preconditioning, *BWR* is the inverse of the scaling factor of memory bandwidth, $N_{ITERlbfgs}$ is the number of iterations to convergence of the GCR with the L-BFGS preconditioner, and $T_{DIAG converge}$ is the time to convergence of the GCR with the diagonal scaling. Using the data in Tables 9, 10 and 11, we obtain *BWR* values in both $100^3$ and $200^3$ cases: 18.5 and 31.6 respectively. Consequently, the L-BFGS preconditioner may converge faster than the diagonal scaling on a device whose memory bandwidth is approximately 20 times lower than that of the device used in the present study. Obviously, the higher bandwidth provides the faster computation. Therefore we can expect that L-BFGS preconditioner performs well on future devices as well as 10-year-old devices in 2016 with the configuration of the present study.

#### Convergence properties

One can show that BFGS–GCR always converges for SPD matrices which are mainly discussed in the present study: First, the residual norm in the flexible GCR always becomes small at each GCR iteration with SPD matrices, and therefore the flexible GCR always converges (Hayami and Sugihara 2004). Second, if the initial Hessian matrix is SPD, the BFGS update always provides SPD Hessian matrices (Nocedal and Wright 1999). Both the diagonal scaling and SSOR provide SPD preconditioning matrices if the original coefficient matrices are SPD (Kushida 2015). Therefore, the Hessian matrices in this study are also SPD within SPD problems. Finally, precenditioned matrices with SPD preconditioning matrices are also SPD (Kushida 2015). With those points, BFGS–GCR always converges with the diagonal scaling and SSOR within SPD problems although the preconditioning matrices vary at each restart point. In addition, as discussed in this section, the BFGS preconditioning provides a better approximation as the BFGS step continues. Consequently, BFGS–GCR converges faster than the preconditioned GCR.

## Conclusion

We developed a new Newton–Raphson based preconditioner (NR preconditioner) for a Krylov type solver. The feasibility of the NR preconditioner was examined and it showed better performance than conventional preconditioners (L-BFGS converges when conventional preconditioners fail, and BFGS–SSOR exhibited a convergence rate over 10 times better than DIAG). In addition, through calculating a condition number for preconditioned matrices, the research confirmed that the NR preconditioner is able to improve matrix conditions. Therefore, it is expected that the NR preconditioner will improve the convergence rate of other Krylov type solvers. Because the convergence rate of the quasi Newton–Raphson methods is superlinear, it is also expected that NR preconditioned Krylov solvers (NR–K solvers) perform well for large scale computing. In fact, in the $200^3$ DOF problem that is the largest problem in the present study, BFGS–DIAG requires 30 % fewer iterations than SSOR. Finally, we confirmed that NR preconditioner performs well on GPU (computational time per iteration on GPU is four times better than on CPU), while conventional ones do not in the prior research.

In theory, solving the initial Hessian matrix in NR–K solvers can be done with other preconditioners, e.g. ILU, SAI, and the multigrid, thus looking for other choices and investigating the peformance are future works.
